# Estimate General Practitioners Active Supply in Iran: Capture-Recapture Method for Three Data Sources

**Published:** 2019-12

**Authors:** Azad SHOKRI, Ali AKBARI-SARI, Mahboubeh BAYAT, Mahmoud KHODADOST, Abbas RAHIMI FOROUSHANI, Elmira MIRBAHAEDDIN, Fereshteh FARZIANPOUR

**Affiliations:** 1. Social Determinants of Health Research Center, Research Institute for Health Development, Kurdistan University of Medical Sciences, Sanandaj, Iran; 2. Department of Health Management and Economics, School of Public Health, Tehran University of Medical Sciences, Tehran, Iran; 3. Gerash University of Medical Sciences, Gerash, Iran; 4. Center for Health Human Resources Research Studies, Ministry of Health and Medical Education, Tehran, Iran; 5. Department of Epidemiology, School of Public Health, Shahid Beheshti University of Medical Sciences, Tehran, Iran; 6. Department of Epidemiology, School of Public Health, Iran University of Medical Sciences, Tehran, Iran; 7. Department of Health Management and Economics, School of Public Health, Tehran University of Medical Sciences, Tehran, Iran; 8. Telfer School of Management, University of Ottawa, Ontario, Canada

**Keywords:** General practitioner, Capture-recapture, Supply, Iran

## Abstract

**Background:**

Accurate estimation of active general practitioners (GPs) is a concern for health authorities to estimate requirements. This study aimed to accurately estimate GPs active supply in Iran using three sources capture-recapture (CRC) method.

**Methods:**

This cross-sectional study collected data during 2015–2016, targeting all GPs registered in three independent data sources; a national survey from all hospitals, database of human resource management office at health ministry and physicians’ offices databank. Variables including medical council codes, GP names, surnames and national ID codes were used for data linkage among the three sources. Three sources CRC method was applied using log-linear models to estimate the total number of active GPs in STATA software.

**Results:**

Overall, 27,048 GPs were identified after removing the duplicate records. Based on CRC three sources data, the total number of GPs were 53,630 in 2015–2016. Distribution of GPs per 1,000 population among the provinces indicates that provinces of Kohgiluyeh & Boyer Ahmad, Mazandaran, Golestan and Yazd with ratios of 1.28, 1.28, 1.21 and 1.17 physicians rank the highest proportion of GPs and the provinces of Sistan & Baluchestan, Ilam, Zanjan, Alborz, North Khorasan with corresponding ratios of 0.24, 0.40, 0.40, 0.43 and 0.45 GPs ranked the lowest.

**Conclusion:**

CRC method is known to be the best and rapidest method to estimate active GP due to its compatibility for the current situation of databanks in Iran. Therefore, this method is a good application in human resource distribution and planning.

## Introduction

General practitioners (GP) are the first providers of medical services for a community and as referral agents they have a significant role in creation of community satisfaction (1). Therefore, the gate keeping role of a GP is one of the most important criteria for a comprehensive and strong healthcare system (2).

However, in case of Iran, there is no accurate estimate of active number of GPs due to lack of a centralized and updated registration system. lack of knowledge about the number of active GPs and existence of contradictory statistics in the early years of implementing family physician program in Iran has created concerns about its success (3, 4). These concerns have raised questions about increase in GPs unemployment due to unrealistic statistics of 80 thousand GPs in the national Medical Council Bank. Moreover, implementation of family physician plan seemed impossible thanks to “misleading statistics” of other data banks underestimating the actual number of active physicians required to fulfill the plan (4, 5). The main reason for this “Bubble Hit” might be incomprehensive detection and registration of active physicians or lack of information about dynamics of GPs.

According to a few existing studies (5, 6), a large number of GPs registered in the medical council databank have chosen to engage in other activities such as administrative or managerial duties at the ministry headquarter or they have left medical practice due to migration, pursuing studies for specialization, retirement or irrelevant fields to medicine such as medical equipment, pharmaceutical, beauty and fitness care and etc. (6, 7). However, due to lack of one centralized and updated registration system, there is no confidence in such reports and therefore, in terms of having accurate information, it has become a serious issue for planners and policy makers. In developed countries, active physicians are estimated through surveys and data matching models because of having valid data banks. In two studies (8, 9) databanks were matched to estimate active number of health workforce while other studies used a combination of matching databanks and conducting surveys (10).

However, databanks of active doctors in Iran are decentralized and they independently relate to different locations. Moreover, they only cover a fraction of active GPs. Recently; capture-recapture (CRC) method has been increasingly favored in various epidemiologic researches (11). Primarily, CRC method was used to estimate animal population (12); then it evolved to other epidemiologic subjects to estimate disease prevalence or any situation in which available data sources are incomplete. Some assumptions have to be considered for this method, for example the sources are supposed independent and all individuals inside the same source have an equal opportunity to be included.

Due to its compatibility with the current situation of databanks in Iran, this study used CRC method as a novel way to estimate active number of GPs and their distribution in the country.

## Materials and Methods

### Three Sources Data Collection

This research was a cross-sectional study conducted in 2015–2016 targeting all GPs in Iran. It utilized data from three independent sources including; a survey among all hospitals in Iran, Ministry of Health and Medical Education (MOHME) database and physicians office data bank from GPs’ licensing office of MOHME. These sources were matched to identify number of active GPs. The national hospitals survey gathered data from 925 hospitals in 2015–2016 using personnel records of each hospital. Human Resources Management (HRM) office in MOHME had a nationally registered database consisting of a list of GPs employed by MOHME. This source is updated on a regular basis upon changes in GPs’ service delivery locations. Physicians’ offices databank was the third nationally registered data source that renews license number of GPs participated in annual trainings for renewal of practice permits and enhancing professional skills in consecutive years. Moreover, data on national population was obtained from Iran’s statistics center.

Three main steps during the national survey of hospitals were data collection, assessment of data quality, and gathering missed data. In the first step, an Excel form together with instruction was sent to the medical universities across the provinces. Requested data on GPs, were about GPs demographic characteristics, national identification code, medical council code, hospital name, province, town. The blank fields of this step were filled by matching medical council national data bank through SQL capacity in Microsoft Office Access. Secondly, quality of the data was evaluated in terms of accuracy. In order to optimize data collection, medical universities introduced focal points as liaisons with the research team to maintain contact on the progress. In case of any issues, the hospitals were reminded by an official letter and direct phone calls to the focal points.

### Linking data among different sources

Medical council code of each GP was used to link the three data sources in SQL capacity of Microsoft Office Access and duplicate records were removed. Afterward, records without a medical council code were sought by matching other variables such as national ID codes, name and last name with medical council reference bank which has all data of medical school graduates. Thereby, the missing medical council codes were extracted and complete.

### Statistical analysis

Three sources CRC method was applied using log-linear models to estimate the total number of active GPs in Iran. Data linkage in previous step identified records of similar variable and it prepared a bank for analysis stage. However, CRC analysis required the following assumptions; data sources were independent and all the GPs had an equal inclusion opportunity (11). Adoption of CRC method had the advantage of more accurate estimation of active GPs, completeness of every data source registries, and finding GPs to population ratio in Iran.

Akaike’s Information Criterion (AIC), Bayesian Information Criteria (BIC), and G2 test also called log likelihood-ratio were used to assess the goodness of fit in model selection. In turn, the best log-linear model with a lower AIC was selected. All statistical calculations were performed in STATA software version 12 (StataCorp, Texas, USA).

## Results

**Fig. 1:**
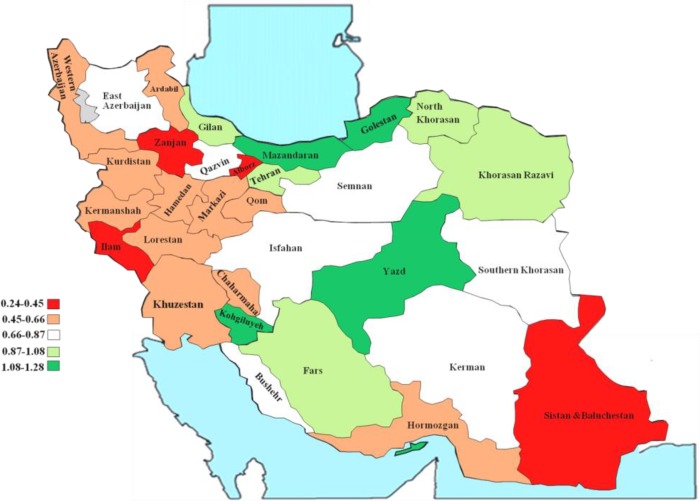
Provincial Distribution of GPs to Populalation Ratio (1,000) in provices of Iran

Overall, 838 hospitals (91%) out of 925 hospitals in Iran participated in the survey. Forty-five (4.86% of) hospitals refused to participate and 42 (4.5% of) hospitals did not answer. Overall, 27,048 GPs were identified after removing duplicate records. A Venn diagram below shows the details of common GPs between the three data sources (Fig. 1).

Data records showed that MOHME HRM office had the largest number of GPs (16,381) while the hospitals survey and the GPs’ licensing office indicated lower numbers 6,986 and 8,837 respectively. 14,609 (54.30% of) GPs were male, the mean age of GPs was 42.24 (±10.32) for men and 37.24 (±8.83) for women. Public sector GPs comprise 69% of all data and as Table 1 shows demographic characteristics, majority of the reported GPs (40%–60%) were in ages 45 to 55.

**Table 1:** Charasteistics of GPs based on three independent sources in 2016

***Variable***		***Hospital survey***	***(A****)*	***MOHME***	***(B)***	***Office***	***(C)***	
		***No***	***%***	***No***	***%***	***No***	***%***	
Work Place by sector							
	Public	5,913	85	16,381	100	0	0
	Private	1,073	15	0	0	8,837	100
Sex							
	Male	4,536	65	7,340	45	6,360	72
	Female	2,450	35	9,041	55	2,477	28
Age							
	40>	2,636	38	9,005	55	689	8
	40–45	1,013	15	1,957	12	1,179	13
	45–55	2,769	40	4,762	29	5,234	59
	55–65	370	5	595	4	1,253	14
	65<	64	1	26	0	482	6

### GPs Supply Estimation Using CRC Method

The best log-linear model with three two-way interactions between resources (C & A, C & B, A & B) were selected based on lower AIC and BIC values, 83.89 and 83.51 respectively (Table 2). Total estimated number of GPs in 2015–2016 was 53,630 (95% CI: 51,578–55,899) while 21,429 GPs were estimated to be missed, i.e. they are not included in any of the three data sources.

**Table 2:** Model selection in log-linear analysis by AIC, BIC and G^2^ statistics

***Model***	***X ^c^***	***N ^d^***	***95% CI for N***	***DF ^b^***	***G^2^^a^***	***BIC ^a^***	***AIC ^a^***
^✦^C/A/B	16353.43	48552.43	(48070.6–49053.02)	4	1617.56	1695.22	1695.44
CA/B	10053.04	42254.04	(41836.59–42689.56)	5	432.50	512.11	512.38
CB/A	16553.54	48754.54	(48214.08–49313.25)	5	1614.71	1694.32	1694.59
AB/C	25846.32	58047.32	(57057.13–59076.96)	5	317.41	397.01	397.28
CA/CB	9115.104	41316.1	(40896.4–41756.07)	6	350.64	432.19	423.52
CA/AB	16103.76	48304.76	(47267.88–49412.98)	6	53.88	135.43	135.75
CB/AB	32198.57	64399.57	(66034.29–62843.86)	6	83.11	164.66	164.99
CA/CB/AB	21428.8	53629.8	(51577.63-55899.32)	7	0.01	83.51	83.89

a Akaike’s Information Criterion/Bayesian Information Criterion/Goodness of fit

b Degree of freedom

c The estimated number of physicians that were not recorded in any of three sources

d The estimated total number of physicians in Iran in 2015

e Pearson Chi^2^ for goodness of fit

✦ C: continuing medical education source. A: hospitals survey Source. B: MOHME HR bank; Model C/A/B: A model where all available resources are independent; Model CA/B: A model where sources C and A are dependent and independent of the source B; Model CB/A: A model where sources C and B are dependent and independent of the source A; Model AB/C: A model where sources A and B are dependent and independent of the source C; Model CA/CB: A model where two sources C and A and also two sources C and B are mutually interdependent and two sources A and B are independent; Model CA/AB: A model where two sources C and A and also two sources A and B are mutually interdependent and two sources C and B are independent; Model CB/AB: A model where two sources C and B and also two sources A and B are mutually interdependent and two sources C and A are independent; Model CA/CB/AB: A model where all two-way interaction between resources are exist

The estimated number of active GPs in each province is showed in Table 2. About 45% were active in provinces of Tehran, Khorasan Razavi, Fars and Esfahan which are major cores of the country. Only less than 12% of the GPs were in provinces of Ilam, North Khorasan, Zanjan, South Khorasan, Semnan, Chaharmahal & Bakhtiari, Qom, Sistan & Baluchistan, Boushehr, Markazi, Hormozgan, and Kurdistan which are in fact small and disadvantaged provinces of the country (Table 3).

**Table 3:** Estimated number of GPs by log-linear model based on three independent sources in 2016

***Province***	***Reported number of GPs***	***Estimated number of GPs***	***95% CI for Estimated number of GPs***	***Completeness of registration (%)***
Alborz	777	898	(865–1464)	96
Ardabil	427	775	(590–1290)	63
Bushehr	288	585	(376–1714)	56
Chaharmahal & Bakhtiari	321	472	(404–688)	79
East Azarbaijan	1347	2,574	(2182–3216)	61
Fars	1923	4,348	(3647–5425)	54
Gilan	1261	2,250	(2023–2594)	71
Golestan	688	1,895	(1363–2965)	44
Hamedan	508	1,004	(802–1433)	62
Hormozgan	352	752	(532–1376)	55
Ilam	179	217	(212–336)	97
Isfahan	2267	3,769	(3521–4106)	76
Kerman	991	2,054	(1653–2777)	56
Kermanshah	652	1,038	(908–1313)	76
Khorasan Razavi	2187	5,212	(4445–6297)	50
Khuzestan	1217	1,946	(1764–2235)	75
Kohgiloyeh & Boyerahmad	252	815	(368–3549)	34
Kurdistan	426	753	(639–1045)	75
Lorestan	432	928	(699–1449)	56
Markazi	433	740	(663–882)	77
Mazandaran	1547	3,751	(3108–4738)	51
North Khorasan	208	363	(296–548)	71
Qazvin	372	963	(658–1681)	45
Qom	370	550	(482–722)	80
Semnan	274	433	(376–576)	78
Sistan & Baluchestan	477	583	(557–682)	94
Southern Khorasan	231	431	(317–884)	65
Tehran	4945	10,460	(9184–12184)	53
Western Azerbaijan	821	1,468	(1182–2131)	66
yazd	627	1,202	(1016–1544)	66
Zanjan	248	390	(330–562)	77

Comparison of current number of GPs to the estimated ones is shown in Table 3. The greatest difference (more than 60%) between number of current GPs and the estimated ones related to provinces of Kohgiluyeh &Boyer Ahmad, Golestan and Qazvin, while the lowest difference (less than 20%) was amongst the provinces of Alborz, Ilam and Sistan & Baluchistan.

### Geographic Distribution of Active GPs

**Fig. 2:**
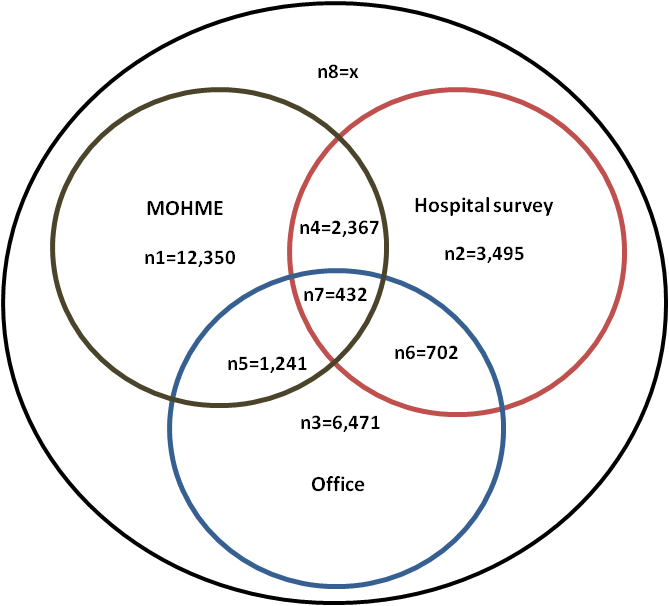
Venn diagram for the common records of GPs between A (Hospital survey), B (MOHME) and C (Office)

Distribution of GPs indicated that provinces of Kohgiluyeh & Boyer Ahmad, Mazandaran and Golestan Yazd with ratios of 1.28, 1.28, 1.21 and 1.17 and Fars, Gilan, Khorasan and Tehran with ratios of 0.92 to 1.00 GPs per 1,000 population had the highest share of GPs. On the contrary, provinces of Sistan & Baluchestan, Ilam, Zanjan, Alborz, North Khorasan with ratio of 0.24, 0.40, 0.40, 0.43 and 0.45 GPs ranked the lowest. Figure 2 elaborates more on the ranking of provinces.

## Discussion

This study aimed to achieve a more accurate estimate of active general practitioners and their distribution across Iran. Overall, 53,630 GPs were active in the country and the existing data banks only showed approximately 50% of the active GPs. Many GPs including those active in the private sector; offices, clinics and other governmental sectors were not registered due to lack of a centralized and up-to-data registration system. The medical council showed 80 thousand record as the most comprehensive existing database for the clinical health workforce, especially for GPs (4, 5). Current estimates showed a 40 thousand difference in number of GPs owing to lack of knowledge on outflow dynamics of GPs because of immigration, unemployment or employment in irrelevant sectors or fields during the past years. The focus has been on inflow of workforce after graduation and changes have not been applied. Due to above limitations mentioned for medical council bank, it might be inappropriate to show GPs distribution in Iran. Therefore, findings of earlier studies using medical council bank should be considered with cution. Furthermore, studies not appling medical council bank presented no comprehensive information because other banks such as human resource bank contain no complete data about whole number of GPs. For example, two studies (13, 14) that used medical council bank and static center data showed only distribution of public GPs. In addition, another study considered only GPs working on the ministry of health (12).

Estimated results of this study show 0.76 GPs per 1,000 population. This ratio in South-East Asia Region (SEARO); Bangladesh, Bhutan, Indonesia, Nepal, Timor-Leste were 0.41, 0.12, 0.11, 0.09, 0.07 respectively and they are significantly lower than Iran (15–18). Ratios of health professionals were lower in Southeast Asia and it has nearly 40% of diseases burden considering failure of preventive plans for communicable diseases (19, 20). In Nordic countries; Scotland, Greenland, Finland, Norway, ratios were 0.84, 0.93, 0.97, 0.92 in order (21) while they were 1.14, 1.15, 0.84, 1.39, 0.91 in Canada, Australia, New Zealand, France and Italy respectively (22, 23). Although they had a significantly higher ratio of GP to population in primary care, these countries face the challenge of an aging population and reducing duration of hospitalization due to development level and better economic status. Nonetheless, in some developed countries such as Germany, Sweden, England, Netherlands, and Denmark (22, 24, 25) this ratio was lower (0.51, 0.60, 0.67, 0.73 and 0.76) and it was 0.70 in Turkey (26). In these countries, some primary care services were delivered in family health centers by family physicians (27–29) who were specialists; hence there were fewer GPs. Comparison of GPs ratio in Iran showed a relatively good condition as a developing country, but it still had a significant difference with developed countries.

Provincial estimation of GPs revealed imbalanced distribution across the country. Underserved provinces of western and south eastern areas had lower ratios similar to South East Asia. In these areas of Iran including Hormozgan province, burden of disease was higher and there was a higher need for healthcare services (30), whereas in advantaged areas, in central and northern regions, this ratio was higher and similar to developed countries and in some case even higher than Nordics countries. Therefore, distribution imbalance has resulted in surplus in advantaged areas with lower need while despite high demand in underserved areas, they had to cope with shortage of skilled GPs (31). This imbalance depends on factors such as financial incentives, life quality and other personal preferences (32). According to WHO, equity in service delivery will be challenged when countries with the lowest need have the most health workers, while those with the greatest burden of disease had the least number (33).

## Conclusion

We noticed a significant difference in number of active GPs in Iran and developing countries. Regarding specialists’ shortage, it is necessary to have an estimate of potential GPs for education in residency. In addition, quantity of health workforce in a country is as important as their distribution amongst different regions considering their demographic, epidemiologic and pathologic characteristics. Therefore, absolutely essential to adopt new approaches for accurate estimation of GPs requirement in national scope and their distribution into underserved areas which suffer from shortage of GPs and greater demand for health services.

## Ethical considerations

Ethical issues (Including plagiarism, informed consent, misconduct, data fabrication and/or falsification, double publication and/or submission, redundancy, etc.) have been completely observed by the authors.
